# Complete Blood Count (CBC)-Derived Inflammation Indexes Are Useful in Predicting Metabolic Syndrome in Adults with Severe Obesity

**DOI:** 10.3390/jcm13051353

**Published:** 2024-02-27

**Authors:** Alice Marra, Adele Bondesan, Diana Caroli, Alessandro Sartorio

**Affiliations:** 1 IRCCS Istituto Auxologico Italiano, Experimental Laboratory for Auxo-endocrinological Research, 28824 Piancavallo-Verbania, Italy; a.bondesan@auxologico.it (A.B.); d.caroli@auxologico.it (D.C.); sartorio@auxologico.it (A.S.); 2 IRCCS Istituto Auxologico Italiano, Experimental Laboratory for Auxo-endocrinological Research, 20145 Milan, Italy

**Keywords:** severe obesity, metabolic syndrome, biomarkers, adults, blood cell count, high-density lipoprotein cholesterol, cardiovascular risk

## Abstract

**Background:** Metabolic syndrome (MetS) is a globally increasing pathological condition. Recent research highlighted the utility of complete blood count-derived (CBC) inflammation indexes to predict MetS in adults with obesity. **Methods:** This study examined CBC-derived inflammation indexes (NHR, LHR, MHR, PHR, SIRI, AISI, and SII) in 231 adults with severe obesity (88 males, 143 females; age: 52.3 [36.4–63.3] years), divided based on the presence (MetS+) or absence (MetS-) of MetS. The relationships between the indexes and the cardiometabolic risk biomarkers HOMA-IR, TG/HDL-C, and non-HDL-C were also evaluated. **Results:** Individuals with metabolic syndrome (MetS+) had significantly higher values of MHR, LHR, NHR, PHR, and SIRI than those without (MetS-) (MHR and NHR: *p* < 0.0001; LHR: *p* = 0.001; PHR: *p* = 0.011; SIRI: *p* = 0.021). These values were positively correlated with the degree of MetS severity. Logistic regression (MHR and NHR: *p* = 0.000; LHR: *p* = 0.002; PHR: *p* = 0.022; SIRI: *p* = 0.040) and ROC analysis (MHR: AUC = 0.6604; LHR: AUC = 0.6343; NHR: AUC = 0.6741; PHR: AUC = 0.6054; SIRI: AUC = 0.5955) confirmed the predictive potential of CBC-derived inflammation indexes for MetS in individuals with severe obesity. CBC-derived inflammation indexes also correlated with HOMA-IR (MHR, LHR, and NHR: *p* < 0.0001; PHR: *p* < 0.001; SIRI: *p* = 0.000) and TG/HDL-C (MHR, LHR, NHR and PHR: *p* < 0.0001; SIRI: *p* = 0.006). **Conclusions:** In conclusion, this study validates CBC-derived inflammation indexes for predicting MetS in individuals with severe obesity. The relationships between these indexes and cardiometabolic risk factors can enable clinicians to better grade MetS associated with obesity.

## 1. Introduction

Metabolic syndrome (MetS) is a disorder often associated with obesity and has become worldwide a major public health concern [1,2,3,4]. It is characterized by various conditions, such as abdominal obesity, hypertension, increased fasting glucose, elevated triglyceride levels, and reduced HDL cholesterol levels [5,6]. Patients with metabolic syndrome are at a higher risk of developing severe pathologies, such as cardiovascular diseases, type 2 diabetes, and non-alcoholic fatty liver disease [7,8,9,10].

It has been widely shown that metabolic syndrome associated with obesity can be considered a chronic state of inflammation, during which visceral adipocytes and macrophages continuously release chemoattractants and cytokines, establishing a positive feedback loop [11,12,13,14]. This condition can accelerate early atherosclerotic changes through several mechanisms, including insulin resistance and inflammation [8].

Furthermore, low levels of high-density lipoprotein cholesterol (HDL-C), which is commonly seen in individuals with MetS, can accelerate the proinflammatory and prothrombotic states during atherogenesis [15,16]. In a physiological state, HDL-C has a protective and antioxidant role, inhibiting cytokine-induced expression of endothelial cell adhesion molecules [17,18]. However, in individuals with low HDL-C levels, this protective effect is diminished, thus leading to an increase in inflammation and atherosclerosis [19].

In the past few decades, increased levels of inflammatory biomarkers (e.g., Interleukin-6, Tumor Necrosis Factor-alpha, C-reactive protein, the neutrophil to lymphocyte ratio, and the platelet to lymphocyte ratio) have been correlated with both obesity and MetS [20,21,22,23,24]. Furthermore, new hematological parameters related to HDL-C and complete blood cell profiles have been proposed as novel inflammatory biomarkers [25,26,27,28,29,30]. These include the monocyte/HDL-C ratio (MHR), lymphocyte/HDL-C ratio (LHR), neutrophil/HDL-C ratio (NHR), platelet/HDL-C ratio (PHR), System Inflammation Response Index (SIRI), Systemic Immune-Inflammation Index (SII), and Aggregate Index of Systemic Inflammation (AISI). Several studies proposed to analyze the potential of these biomarkers in predicting MetS in individuals with obesity and their correlation with cardiometabolic risk factors [31,32,33,34,35,36,37,38,39]. However, to the best of our knowledge, no studies assessed MHR, LHR, NHR, PHR, SIRI, SII, and AISI data in relation to MetS in patients with severe obesity. To address this gap, this study was designed to understand the relationships between inflammation biomarkers derived from complete blood count (CBC) and the occurrence of metabolic syndrome in adults with severe obesity.

## 2. Material and Methods

### 2.1. Patients

The study cohort comprised 231 adults diagnosed with severe obesity, consisting of 88 males and 143 females, with a median age (interquartile range) of 52.3 [36.4–63.3] years and a median BMI (interquartile range) of 44.2 [40.4–46.1] years. These individuals were admitted to the Division of Metabolic Diseases at IRCCS Istituto Auxologico Italiano, Piancavallo-Verbania, Italy, to undergo a three-week multidisciplinary integrated body weight reduction program (BWRP), entailing integrated energy-restricted diet, physical rehabilitation (moderate aerobic activity), psychological counseling, and nutritional education [40]. A Mediterranean diet was prescribed based on the basal metabolic rate and physical activity level of each patient. Energy intake was calculated by subtracting approximately 500 kcal from the resting energy expenditure measurement. The macronutrient composition of the diet included 21% proteins, 53% carbohydrates, and 26% lipids. Daily estimated water intake was set at 1000 mL, while the estimated salt intake was 1560 mg Na^+^, 3600 mg K^+^, and 900 mg Ca^2+^. Patients were encouraged to consume at least 2000 mL of additional water per day.

The physical activity regimen consisted of five days of training per week, including one hour of dynamic aerobic standing and floor exercises involving arms and legs, guided by a therapist, and either 20–30 min of cycling at 60 W or 3–4 km of outdoor walking on flat terrain, based on individual capabilities and clinical status.

The psychological counseling program consisted of two or three sessions per week of individual and/or group psychotherapy conducted by clinical psychologists. Furthermore, lectures on obesity-related issues and risks, motivational talks, demonstrations of healthy food choices, food preparation workshops, and group discussions, with or without supervision took place daily.

Obesity was defined in adulthood as the presence of a BMI >30 kg/m^2^. Exclusion criteria were acute or chronic kidney/liver diseases, secondary obesity, acute infection or inflammatory conditions, autoimmune diseases, malignant diseases, neurodegenerative diseases, and hematological and/or oncological disorders.

For each participant, anthropometric and instrumental measurements and metabolic variables were collected.

The NHR, MHR, LHR, PHR, SII, SIRI, and AISI were calculated using the following formulas:

NHR = neutrophil (10^9^/L)/HDL-C (mg/dL) ratio; MHR = monocyte (10^9^/L)/HDL-C (mg/dL); LHR = lymphocyte (10^9^/L)/HDL-C (mg/dL) ratio; PHR = platelet (10^9^/L)/HDL-C (mg/dL) ratio; SII = platelet (10^9^/L)/ × neutrophil (10^9^/L)-to-lymphocyte (10^9^/L) ratio, SIRI = monocyte (10^9^/L)/× neutrophil (10^9^/L)-to-lymphocyte (10^9^/L) ratio; AISI = neutrophil (10^9^/L)/× platelet (10^9^/L)/ × monocyte (10^9^/L)-to-lymphocyte (10^9^/L) ratio.

The cardiovascular risk indexes were calculated as follows: HOMA-IR = fasting insulin (mU/L) × fasting glucose (mmol/L)/22.5; TG/HDL-C ratio = total triglycerides (mmol/L)/HDL-C (mg/dL); non-HDL-C = total cholesterol (mg/dL) − HDL-C (mg/dL).

### 2.2. Metabolic Syndrome Definition

According to the IDF criteria for the diagnosis of metabolic syndrome [41,42], adult patients with obesity were identified as having metabolic syndrome if they had three or more of the following altered factors (defined as MetS criteria in this study):

(i) Abdominal obesity (WC ≥ 102 cm for males; ≥88 cm for females);
(ii) Elevated triglycerides: ≥150 mg/dL (1.7 mmol/L) or specific treatment for this lipid abnormality;
(iii) Reduced HDL-C: <40 mg/dL (1.0 mmol/L) in males; <50 mg/dL (1.3 mmol/L) in females or specific treatment for this lipid abnormality;
(iv) Increased BP: SBP ≥ 130 mmHg or DBP ≥ 85 mmHg and/or treatment of previously diagnosed hypertension;
(v) Increased fasting plasma glucose (FPG) concentration ≥100 mg/dL (5.6 mmol/L) or previously diagnosed type 2 diabetes mellitus.

Patients were subsequently subdivided into three subgroups according to the number of identified MetS criteria: MetS 1–2 (i.e., the presence of 1–2 MetS criteria = free from MetS), MetS 3 (i.e., the presence of 3 MetS criteria), and MetS 4–5 (i.e., the presence of 4–5 criteria). Patients in subgroups MetS 1–2 were considered MetS-, while those in subgroups MetS 3 and MetS 4–5 were considered MetS+.

### 2.3. Anthropometric Measurements

The physical examination included the evaluation of height, weight, and waist circumference (WC) by trained operators following the Anthropometric Standardization Reference Manual [43]. Height measurements were taken using a Harpenden Stadiometer (Holtain Limited, Crymych, SA41 3UF, UK) while body weight was recorded to the nearest 0.1 kg using an electronic scale (Ro WU 150, Wunder Sa.bi., Trezzo sull’Adda, 20056 Milan, Italy). Waist circumference was measured with a non-elastic flexible tape measure in a standing position, midway between the lowest rib and the top of the iliac crest, following gentle expiration [44].

### 2.4. Laboratory Analyses

Approximately 10 mL of blood samples were obtained from each participant in standard tubes at 8:00 AM following an overnight fast. Subsequently, blood count and metabolic variables were assessed.

Hematologic parameters were analyzed using Beckman Coulter instruments. Leukocyte count was performed with the impedance-based method upon erythrocyte (RBC) lysis. The determination of leukocyte subtype populations relied on the assessment of volume, conductivity, and scatter properties of leukocytes (VCS Technology).

Colorimetric enzymatic assays (Roche Diagnostics, Monza, Italy) were used to determine serum HDL-C and triglyceride levels. Serum glucose level was measured by the glucose oxidase enzymatic method (Roche Diagnostics, Monza, Italy). All serum analyses for the determination of HDL-C, triglycerides, and glucose were performed by using a Roche Cobas 6000 analyzer.

### 2.5. Blood Pressure Measurement

Blood pressure (BP) was measured in subjects in a sitting position and a relaxed condition using a sphygmomanometer with a properly sized cuff placed on the right arm at rest. The procedure was repeated three times, and the averages of the three values for systolic and diastolic BP were recorded

### 2.6. Statistical Analysis

GraphPad Prism 10.2.0 software for Windows (GraphPad Software, San Diego, CA, USA, https://www.graphpad.com/ accessed on 8 August 2023) was used to perform the analysis and for data plotting.

**Figure 1:**
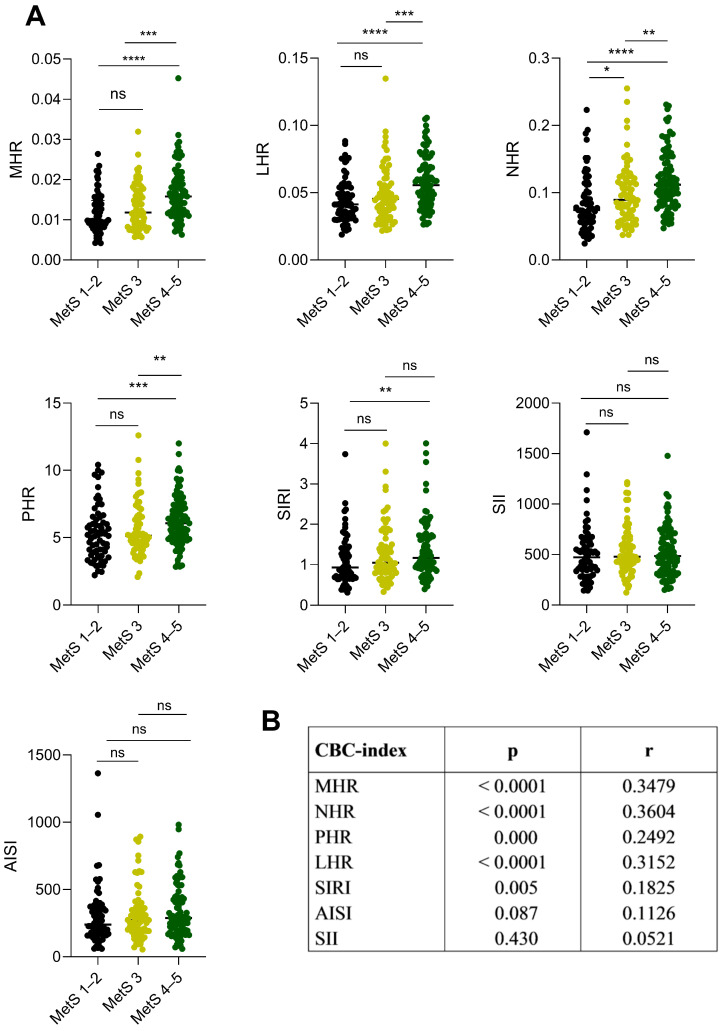
CBC-derived inflammation indexes in relation to the severity of the metabolic syndrome (MetS 1–2, MetS 3, and MetS 4–5 altered factors) in patients with severe obesity. (**A**) Dot plots represent the CBC-derived inflammation indexes with the severity of metabolic syndrome. The amount of IDF criteria indicates the severity of metabolic syndrome: MetS 1–2 (low grade, no metabolic syndrome in black), MetS 3 (moderate grade, in light green), MetS 4–5 (high grade in dark green). The difference between the MetS subgroups was calculated for each index using the non-parametric Mann–Whitney U test. * = *p* ≤ 0.05; ** = *p* ≤ 0.01; *** = *p* ≤ 0.001; **** = *p* ≤ 0.0001; ns (not significant) = *p* > 0.05. The *p*-value (*p*) was considered significant when ≤ 0.05. (**B**) The non-parametric Spearman’s rank correlation test revealed a positive correlation between CBC-derived inflammation indexes and metabolic syndrome severity. Abbreviations: r correlation coefficient; *p p*-value. Abbreviations: CBC-index complete blood count-derived inflammation index; MHR monocyte-to-HDL-C ratio; LHR lymphocyte-to-HDL-C ratio; NHR neutrophil-to-HDL-C ratio; PHR platelet-to-HDL-C ratio; SII Systemic Immune Inflammation Index; SIRI Systemic Immune Response Index; AISI Aggregate Index of Systemic Inflammation. The *p*-value (*p*) was considered significant when ≤ 0.05. The interval confidence considered was 95%.

**Figure 2:**
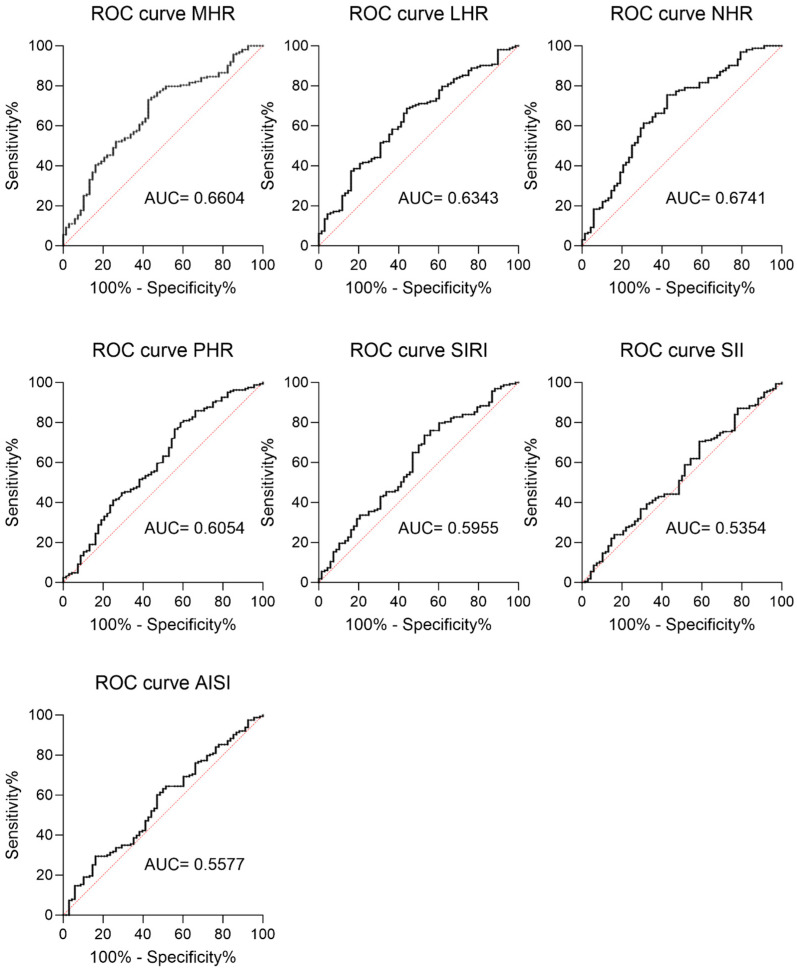
ROC curve of CBC-derived inflammation indexes as a prediction marker for metabolic syndrome in individuals with severe obesity. ROC (receiver operating characteristic) curves for all the analyzed CBC-derived inflammation indexes are reported. MHR: AUC = 0.6604 (95% CI 0.5838 to 0.7370, sensitivity 73.01%, specificity 70.59%, *p* = 0.000); LHR: AUC = 0.6343 (95% CI 0.5569 to 0.7118, sensitivity 70.55%, specificity 67.65%, *p* = 0.001); NHR: AUC = 0.6741 (95% CI 0.5958 to 0.7525, sensitivity 67.48%, specificity 70.59%, *p* < 0.0001); PHR: AUC = 0.6054 (95% CI 0.5226 to 0.6882, sensitivity 60.12%, specificity 64.71%, *p* = 0.012); SIRI: AUC = 0.5955 (95% CI 0.5140 to 0.6771, sensitivity 59.51%, sensibility 63.24%, *p* = 0.02); SII: AUC = 0.5577 (95% CI 0.4766 to 0.6387, sensitivity 55.21%, sensibility 58.82%, *p* = 0.167); and AISI: AUC = 0.5364 (95% CI 0.4541 to 0.6186, sensitivity, 53.37% sensibility 57.35%, *p* = 0.384).

Each variable was tested for normal distribution and linearity using the Shapiro–Wilk normality test. Due to the lack of normalcy, descriptive statistics of the continuous and categorical variables in Table 1, Table 2, Table 3 and Table 4 and Figure 1 and Figure 2 were reported as median (interquartile range) or percentage.

Each parameter was evaluated as a continuous variable and compared among the subgroups (obese MetS-/obese MetS+, MetS severity grades) using the non-parametric Mann–Whitney U test, except for sex, which was considered a categorical variable. A two-way ANOVA was used to assess how sex affects MetS prevalence. Fisher’s exact test was used to compare contingency tables and categorical variables.

The non-parametric Spearman’s rank correlation test was used to assess the correlation between the increase in CBC-derived inflammation indexes and metabolic syndrome severity grade, as well as cardiometabolic risk factors.

To determine the effect of the CBC-derived inflammation indexes as predictors (considered as independent variables) of the metabolic syndrome, binary logistic regression analysis was used.

ROC (receiver operating characteristic) curves were used to demonstrate the ability to distinguish between MetS- and MetS+. Graphs were drawn using raw data for the CBC-derived inflammation indexes of MetS- and MetS+, and the area under the ROC curves (AUC) was used to assess the accuracy of those indexes. The optimal CBC-derived inflammation indexes cut-off, the values of area under the curve (AUC), and the sensitivity and specificity for the development of MS were calculated using the same data.

A two-tailed *p*-value ≤ 0.05 was considered significant for all data analyses. The confidence interval considered was 95% for all datasets.

A power analysis was carried out before starting the patient selection. A sample size of (at least) 50 subjects for each group (i.e., MetS+ or MetS-) was calculated to be adequate to evidence a statistically significant mean difference of 0.004 for MHR, with a standard deviation of 0.07 (for both groups), applying a *t*-test with a power of 80% and an α error of 0.05.

## 3. Results

A total of 231 patients (88 males and 142 females) with severe obesity were included in this retrospective study. The median BMI (interquartile range) was 43.6 kg/m^2^ (from 40.5 to 48.3) and the median age was 52.3 years (interquartile range 36.4–63.3). The clinical and biochemical data are shown in Table 1. The study population was divided into two subgroups based on the IDF criteria for the definition of MetS: those who met the criteria (MetS+, N = 163, 70.6%) and those who did not (MetS-, n = 68, 29.4%). Among males, 71 (81%) were considered affected by the syndrome, while MetS was present in 92 females (64%).

**Table 1:** Main characteristics of the study group (whole study population, obese without MetS and with MetS).

Parameters	All Obese (N = 231)	Obese MetS- (N = 68, 29.4%)	Obese MetS+ (N = 163, 70.6%)	*p* MetS- vs. MetS+
Age (years)	52.3 (36.4–63.3)	44.9 (27.3–62.4)	52.9 (41.5–63.7)	0.027
Sex (N, %)	M 88, 38; F 143, 62	M 17, 19; F 51, 36	M 71, 81; F 92, 64	0.942
BMI (kg/m^2^)	43.6 (40.5–48.3)	42.7 (40.4–46.1)	44.2 (40.8–48.9)	0.052
WC (cm)	121.0 (114.0–132.0)	115.0 (108.0–126.3)	125.0 (117.0–134.0)	<0.0001
SBP (mmHg)	135.0 (120.0–145.0)	125.0 (120.0–140.0)	140.0 (130.0–150.0)	<0.0001
DBP (mmHg)	80.0 (80.0–90.0)	80.0 (80.0–80.0)	80.0 (80.0–90.0)	0.002
TG (mmol/L)	130.0 (102.0–169.0)	104.5 (89.2–129.8)	145.0 (114.0–186.0)	<0.0001
FBG (mmol/L)	5.4 (4.9–6.1)	5.0 (5.3–4.6)	5.8 (6.8–5.2)	<0.0001
HDL-C (mg/dL)	44.0 (38.0–53.0)	51.5 (43.0–59.0)	42.0 (36.0–49.0)	<0.0001
Total Cholesterol (mg/dL)	183.0 (155.0–210.0)	179.5 (154.0–199.8)	183.0 (156.0–214.0)	0.242

WC, SBP, DBP, TG, FBG, and HDL are used as criteria by the IDF for the diagnosis of metabolic syndrome. Abbreviations: MetS- patients with severe obesity without metabolic syndrome; MetS+ patients with severe obesity and metabolic syndrome; M males; F females; BMI body mass index in kg/m^2^; WC waist circumference in cm; SBP systolic blood pressure in mm/Hg; DBP diastolic blood pressure in mm Hg; TG triglyceride in mmol/L; FBG fasting blood glucose in mmol/L; HDL-C high-density lipoprotein in mmol/L. The non-parametric Mann–Whitney U test was used to show the difference between MetS- and MetS+. Fisher’s exact test was used to determine the effect of gender on the prevalence of MetS (*p* = 0.942). The data are presented as median (interquartile range) or percentage (%). A *p*-value (*p*) ≤ 0.05 is considered statistically significant.

In both genders, the MetS+ group had significantly higher BMI, triglycerides (TGs), waist circumference (WC), fasting blood glucose (FBG), and systolic (SBP) and diastolic (DBP) blood pressure compared with the MetS- group (BMI: *p* = 0.052; TG: *p* < 0.0001; WC *p* < 0.0001; FBG: *p* < 0.0001; SBP: *p* < 0.0001; DBP: *p* = 0.002). Conversely, the MetS+ group had significantly lower serum HDL levels than the MetS- group (*p* < 0.0001). Total cholesterol levels were comparable between the two groups (*p* = 0.242).

Based on the IDF’s age-specific criteria for defining metabolic syndrome, a significantly higher prevalence of subjects with MetS was found in the male compared with the female population (*p* < 0.0001).

Despite the difference in prevalence of males and females in the whole population, gender was not a factor discriminating the different distribution of MetS (*p* = 0.942).

In the whole study group, the most frequently altered parameters were WC (231 out of 231 patients, 100%), followed by BP (179/231, 77.5%) and HDL-C (125/231, 54.1%), while FBG was altered in 111/231 patients (48.1%) and TG in 84/231 patients (36.4%). The MetS+ population was further divided into three subgroups based on the number of altered criteria for the presence of MetS. These subgroups were named MetS 1–2, MetS 3, and MetS 4–5 depending on whether they had one to two (N = 68, 29.4%), three (N = 72, 31.2%), or four to five altered criteria (N = 91, 39.4%), respectively.

Total white blood cell count, CBC-derived inflammation indexes, and cardiometabolic biomarkers in the whole study population, and in the subgroups with (MetS+) and without MetS (MetS-), are shown in Table 2.

**Table 2:** Hematologic parameters of the study group (whole study population, obese without MetS and with MetS).

Parameters	All Obese (N = 231)	Obese MetS- (N = 68; 29.4%)	Obese MetS+ (N = 163; 70.6%)	*p* MetS- vs. MetS+
Leukocytes (10^9^/L)	7.2 (6.1–8.5)	6.8 (5.9–8.4)	7.3 (6.2–8.5)	0.059
Neutrophils (10^9^/L)	4.1 (3.4–5.0)	3.7 (3.2–4.7)	4.3 (3.5–5.2)	0.023
Lymphocytes (10^9^/L)	2.1 (1.7–2.5)	2.0 (1.8–2.5)	2.2 (1.7–2.6)	0.946
Monocytes (10^9^/L)	0.6 (0.5–0.7)	0.5 (0.5–0.7)	0.6 (0.5–0.7)	0.202
Eosinophils (10^9^/L)	0.2 (0.1–0.2)	0.2 (0.1–0.2)	0.2 (0.1–0.3)	0.974
Basophils (10^9^/L)	0.0 (0.0–0.1)	0.0 (0.0–0.0)	0.0 (0.0–0.1)	0.156
Platelets (10^9^/L)	250.0 (206.0–290.0)	256.0 (209–302.0)	247.0 (202.0–283.0)	0.131
MHR	0.013 (0.009–0.018)	0.010 (0.008–0.015)	0.014 (0.011–0.019)	<0.0001
LHR	0.05 (0.04–0.06)	0.04 (0.03–0.05)	0.05 (0.04–0.07)	0.001
NHR	0.09 (0.07–0.13)	0.07 (0.06–0.11)	0.10 (0.08–0.13)	<0.0001
PHR	5.4 (4.5–7.0)	5.2 (3.8–6.3)	5.6 (4.7–7.1)	0.011
SIRI	1.1 (0.8–1.6)	0.9 (0.7–1.4)	1.1 (0.9–1.7)	0.022
SII	477.5 (344.1–665.3)	471.5 (338.3–628.3)	478.6 (355.2–668.7)	0.385
AISI	269.5 (184.7–401.5)	238.9 (169.3–364.1)	273.1 (191.4–423.0)	0.169
HOMA–IR	4.8 (3.1–7.0)	3.4 (2.3–4.8)	5.1 (3.7–8.1)	<0.0001
Non-HDL-C	138.0 (110.0–164.0)	126.0 (107.3–146.8)	142.0 (114.0–166.0)	0.008
TG/HDL-C	3.0 (2.1–4.2)	2.1 (1.6–2.6)	3.4 (2.5–4.8)	<0.0001

Abbreviations: MetS- patients with severe obesity without metabolic syndrome; MetS+ patients with severe obesity and metabolic syndrome; MHR monocyte-to-HDL-C ratio; LHR lymphocyte-to-HDL-C ratio; NHR neutrophil-to-HDL-C ratio; PHR platelet-to-HDL-C ratio; SII Systemic Immune Inflammation Index (platelet × neutrophil-to-lymphocyte ratio); SIRI Systemic Immune Response Index (monocyte × neutrophil-to-lymphocyte ratio); AISI Aggregate Index of Systemic Inflammation (neutrophil × platelet× monocyte-to-lymphocyte ratio); HOMA-IR Homeostatic Model Assessment for Insulin Resistance (fasting insulin (mU/L) x fasting glucose (mmol/L)/22.5); non-HDL-C non-HDL cholesterol (total cholesterol − HDL-C; TG/HDL-C triglyceride-to-HDL-C ratio. The difference between the MetS- and MetS+ groups was calculated using the non-parametric Mann–Whitney U test. The *p*-value (*p*) was considered significant when ≤0.05. Data is given as median (interquartile range) or %.

The total leukocyte count was slightly lower in the MetS- population compared with the MetS+ (*p* = 0.059). Specifically, there was no significant difference in the total number of lymphocytes (*p* = 0.911), monocytes (*p* = 0.202), eosinophils (*p* = 0.974), or basophils (*p* = 0.155) between the two subgroups; only the total neutrophil count was significantly higher in MetS+ compared with MetS- (*p* = 0.023). The platelet count did not differ between the two subgroups (*p* = 0.131).

Patients with MetS+ had significantly higher blood-derived inflammatory indexes (Table 2). In particular, the monocyte/HDL-C ratio (MHR), lymphocyte/HDL-C ratio (LHR), neutrophil/HDL-C ratio (NHR), platelet/HDL-C ratio (PHR), and System Inflammation Response Index (SIRI) were significantly higher in patients with MetS than in those without (LHR: *p* = 0.001; NHR: *p* < 0.0001; MHR: *p* < 0.0001; PHR: *p* = 0.011; SIRI: *p* = 0.021). By contrast, no significant differences in the Systemic Immune-Inflammation Index (SII) or the Aggregate Index of Systemic Inflammation (AISI) were found between the two subgroups (SII: *p* = 0.385; AISI: *p* = 0.169).

The HOMA-IR, TG/HDL ratio, and non-HDL-C ratio, which are considered valuable cardiometabolic indexes, were significantly higher in the MetS+ than in the MetS- group (HOMA-IR: *p* < 0.0001; non-HDL-C: *p* = 0.008; TG/HDL: *p* < 0.0001) (Table 2).

The inflammation indexes derived from CBC were analyzed based on the number of altered MetS criteria. The MHR, LHR, NHR, PHR, and SIRI index were higher in MetS 4–5 patients when compared with MetS 1–2 patients (Figure 1A, MHR: *p* < 0.0001; LHR: *p* < 0.0001; NHR: *p* < 0.0001; PHR: *p* = 0.000; SIRI: *p* = 0.009) and with MetS 3 patients (Figure 1A, MHR: *p* = 0.000; LHR: *p* = 0.0008; NHR: *p* < 0.001; PHR: *p* = 0.002), except for the SIRI index, which resulted to be comparable in MetS 3 and MetS 1–2 patients (*p* = 0.251).

No differences were found when comparing the MetS 1–2 and MetS 3 patients (Figure 1A, MHR: *p* = 0.132; LHR: *p* = 0.348; PHR: *p* = 0.449; SIRI: *p* = 0.187), except for the NHR index, which was significantly higher in the MetS 3 than in MetS 1–2 patients (Figure 1A, *p* = 0.037). As expected, no significant differences were observed between the three subgroups when looking at the SII and AISI indexes (Figure 1A, SII: MetS 4–5 vs. MetS 3 *p* = 0.993, MetS 4–5 vs. MetS 1–2 *p* = 0.412, MetS 3 vs. MetS 1–2 *p* = 0.488; AISI: MetS 4–5 vs. MetS 3 *p* = 0.482, MetS 4–5 vs. MetS 1–2 *p* = 0.112, MetS 3 vs. MetS 1–2 *p* = 0.447).

A positive correlation was found between MHR, LHR, NHR, PHR, and SIRI with the number of MetS criteria (MHR: *p* < 0.0001; LHR: *p* < 0.0001; NHR: *p* < 0.0001; PHR: *p* = 0.0001; SIRI; *p* = 0.005), while no correlation was found for SII (*p* = 0.430) and AISI (*p* = 0.087) (Figure 1B).

Evaluation of CBC-derived inflammation indexes and metabolic syndrome was performed using simple logistic regression analysis, which confirmed that the MHR, LHR, NHR, PHR, and SIRI were the only indexes significantly related to MetS (Table 3, MHR: *p* = 0.000; LHR: *p* = 0.002; NHR: *p* = 0.000; PHR: *p* = 0.022; SIRI: *p* = 0.040).

**Table 3:** Binary logistic regression of the association between metabolic syndrome and CBC-derived inflammation indexes.

Independent Variables	OR (95% CI)	*p*
MHR	7.31 (1.53; 3.49)	0.000
LHR	7.13 (21.5; 2.36)	0.002
NHR	18.0 (76.6; 4.61)	0.000
PHR	1.20 (1.02; 1.41)	0.022
SIRI	1.66 (1.02; 2.72)	0.040
SII	1.00 (0.99; 1.00)	0.585
AISI	1.00 (0.99; 1.00)	0.303

Abbreviations: OR: odds ratio; CI: confidence interval; MHR monocyte-to-HDL-C ratio; LHR lymphocyte-to-HDL-C ratio; NHR neutrophil-to-HDL-C ratio; PHR platelet-to-HDL-C ratio; SII Systemic Immune Inflammation Index; SIRI Systemic Immune Response Index; AISI Aggregate Index of Systemic Inflammation. The *p*-value (*p*) was considered significant when ≤ 0.05.

In individuals with MetS, only the MHR, LHR, NHR, PHR, and SIRI showed a positive and significant correlation with the cardiometabolic indexes HOMA-IR and TG/HDL (Table 4, HOMA-IR: MHR: *p* < 0.0001; LHR: *p* < 0.0001; NHR: *p* < 0.0001; PHR: *p* < 0.0001; SIRI: *p* = 0.0004. TG/HDL-C: MHR: *p* < 0.0001; LHR: *p* < 0.0001; NHR: *p* < 0.0001; PHR: *p* < 0.0001; SIRI: *p* = 0.0065). AISI was found to be significantly correlated with the HOMA-IR factor (*p* = 0.0008). No correlation was observed between the non-HDL-C index and all the CBC-derived inflammation indexes (Table 4).

**Table 4:** Correlations between CBC-derived inflammation indexes and cardiometabolic risk biomarkers in individuals with severe obesity.

	HOMA-IR		Non-HDL-C		TG/HDL-C	
CBC-Index	r	*p*	r	*p*	r	*p*
MHR	0.371	<0.0001	−0.038	0.562	0.552	<0.0001
LHR	0.319	<0.0001	0.108	0.099	0.550	<0.0001
NHR	0.317	<0.0001	0.003	0.963	0.543	<0.0001
PHR	0.278	<0.0001	0.020	0.757	0.442	<0.0001
SIRI	0.231	0.000	−0.117	0.075	0.178	0.006
SII	0.114	0.085	−0.072	0.273	0.011	0.856
AISI	0.220	0.000	−0.094	0.153	0.093	0.154

The correlations between cardiometabolic risk biomarkers and CBC-derived inflammation indexes were calculated using the non-parametric Spearman’s test. The MHR, LHR, NHR, PHR, and SIRI were significantly correlated with the HOMA-IR and TG/HDL-C but not with non-HDL-C. No correlation was found for the SII and AISI indexes. Abbreviations: MHR monocyte-to-HDL-C ratio; LHR lymphocyte-to-HDL-C ratio; NHR neutrophil-to-HDL-C ratio; PHR platelet-to-HDL-C ratio; SII Systemic Immune Inflammation Index; SIRI Systemic Immune Response Index; AISI Aggregate Index of Systemic Inflammation; r correlation coefficient. The *p*-value (*p*) was considered significant when ≤ 0.05. The interval confidence considered was 95%.

The correlations between the CBC-derived inflammatory indexes and the waist circumference (WC) and blood pressure values (i.e., the most frequently altered parameters in MetS+ patients in the present study) were also taken into consideration. All the CBC-derived inflammatory indexes were positively correlated with the WC values (MHR, NHR, SIRI: *p* < 0.0001; LHR: *p* = 0.032; PHR: *p* = 0.050; AISI: *p* = 0.007), except for SII (*p* = 0.130). By contrast, no correlation was found between all the CBC-derived inflammation indexes and the blood pressure values (*p* > 0.05).

In addition, a ROC curve analysis was performed to assess the potential predictive role of each inflammatory index in the overall population (Figure 2). The analysis revealed that MHR, LHR, NHR, PHR, and SIRI had significant discriminatory values for MetS (Figure 2, MHR: AUC=0.6604; LHR: AUC= 0.6343; NHR: AUC=0.6741; PHR: AUC= 0.6054; SIRI: AUC=0.5955). By contrast, AISI and SII had a lower predictive power (AISI: AUC=0.557; SII: AUC= 0.5364), and their *p*-values reflected the lack of difference between the MetS+ and MetS- subjects (Figure 2, AISI *p* = 0.291; SII *p* = 0.581).

## 4. Discussion

This study aimed to assess the usefulness of new inflammation indexes derived from complete blood count (CBC) and their relationship with biomarkers of cardiometabolic risk in adult patients with metabolic syndrome (MetS) and severe obesity.

MetS is a complex condition that, especially when associated with obesity, increases the likelihood of developing atherosclerosis, cardiovascular disorders, and metabolic diseases [10]. The underlying mechanism behind the development of severe and chronic disorders in individuals with MetS is strongly linked to the establishment of chronic inflammation, mainly induced by the continuous release of cytokines and adipokines from the adipose tissue, which can recruit white blood cells [11].

The effects of adipokines and cytokines released from adipose tissue have been associated with insulin resistance, which plays an important role in both the development and progression of inflammatory, cardiovascular, and atherogenic processes [13,45,46].

During an inflammatory response, the body activates a series of events involving circulating white blood cells (i.e., neutrophils) [14], which in turn may stimulate the secretion of cytokines and proteolytic enzymes. These molecules can lead to tissue damage by activating the coagulation cascade and disrupting the integrity of the endothelial cells. A decrease in lymphocyte count is often observed during inflammatory conditions, and this has been linked to poor prognosis in coronary artery disease. Monocytes are also activated during inflammation and can trigger the production of inflammatory cytokines.

Moreover, in patients with MetS, the amount of HDL-C is significantly reduced [41]. In health conditions, HDL-C molecules are responsible for removing cholesterol from cells and exerting anti-inflammatory, antioxidant, and antithrombotic effects [17,18]. They are also highly effective in inhibiting the expression of adhesion molecules on endothelial cells, thus preventing monocyte recruitment to the artery wall. Therefore, a lower HDL-C count and higher monocyte count may indicate inflammation [47].

A growing number of studies demonstrated that biomarkers derived from peripheral blood cells, such as the neutrophil to HDL-C ratio [27,36,48], monocyte to HDL-C ratio (MHR) [32,34,49,50], lymphocyte to HDL-C ratio (LHR) [27,35,36,37], platelet to HDL-C ratio (PHR) [27,39,51], System Inflammation Response Index (SIRI) [25,26,31,49,50], Systemic Immune-Inflammation Index (SII) [25,28,31,33], and Aggregate Index of Systemic Inflammation (AISI) [27,52,53], can be used to detect and predict the presence and severity of systemic inflammatory processes, including cardiovascular diseases and MetS.

To date, there is limited evidence on the use of CBC-derived inflammation indexes in adult patients with MetS, and, to the best of our knowledge, no studies have been performed in a population with severe obesity. Most of the published research was in fact developed on individuals with mild obesity (BMI < 35 kg/m^2^), evaluating a limited number of CBC-derived inflammation indexes and thus providing a limited overview of the scenario.

Therefore, in the present study, we assessed for the first time the diagnostic value of the novel inflammatory biomarkers MHR, NHR, PHR, LHR, SIRI, SII, and SIRI derived from peripheral blood cells and HDL-C in people with MetS associated with severe obesity. These parameters were analyzed in a large group of adults with severe obesity (N = 231, BMI >35 kg/m^2^), subdivided into three subgroups (MetS 1–2, MetS 3, and MetS 4–5) based on the altered components determining the MetS presence and severity.

In agreement with previous studies, a significantly higher number of total leukocytes, with a significant increase in neutrophils [20,50,54], was found in our MetS+ population, while no differences were found in the numbers of monocytes, platelets, or lymphocytes.

A significant increase in MHR, NHR, PHR, LHR, and SIRI was detected in MetS+ individuals compared with those MetS-, while no differences were found for the AISI and SII indexes. This finding suggests that the hematological parameters that altered the inflammation state the most are the neutrophils and the HDL-C, the ratio being influenced by the increase in the neutrophil count and the decrease in HDL-C values in MetS+.

Spearman correlation analysis and binary logistic regression showed that all indicators, except for SII and AISI, were positively associated with the presence and severity of MetS, thus showing an increase in terms of inflammation with the worsening of the metabolic syndrome condition.

Additionally, the ROC curve analysis supported these results, showing a significant predicting power for LHR, MHR, NHR, PHR, and SIRI but not for AISI and SII in our MetS+ population.

The most altered parameters found in our MetS+ population were waist circumference (100%), blood pressure (77.4%), and HDL-C (54.1%).

These factors are major determinants for cardiometabolic risk, and the risk is higher in people with obesity during adulthood, especially for those with a greater MetS load [10,55].

Therefore, the correlations between the CBC-derived inflammation indexes and the cardiovascular risk biomarkers were assessed (i.e., HOMA-IR [56,57,58], TG/HDL-C [59,60], and non-HDL-C [61]).

Several studies have highlighted the importance of combined biomarkers, such as the HOMA-IR ratio, TG/HDL-C, and non-HDL-C, in predicting atherogenic lipid profiles and insulin resistance. In our investigation, we found significant positive correlations between LHR, NHR, MHR, PHR, and SIRI with both HOMA-IR and TG/HDL-C. This correlation was probably due to the higher values of fasting insulin and fasting glucose found in MetS+ people. Surprisingly, no correlation between the CBC-derived inflammation indexes and non-HDL-C was found.

Overall, these findings suggest that an elevated inflammation level, indicated by an increase in CBC-derived inflammation index values (LHR, MHR, NHR, and PHR) was associated with a higher risk of cardiovascular diseases, considering HOMA-IR and TG/HDL-C as risk factors.

Several limitations in our study must be taken into consideration.

Firstly, our study lacks longitudinal data, preventing us from discerning the chronological sequence of MetS onset and development and its implications for patient health. Additionally, information regarding the initiation and duration of MetS treatment was not uniformly collected among all participants. Furthermore, despite considering several potential confounding factors, to date, we cannot exclude the possibility that MetS may be influenced by other lifestyle variables that could impact the concentrations of CBC-derived inflammation indexes.

Moreover, the higher prevalence of treated patients with metabolic syndrome could have reduced the difference between both groups for several parameters.

While our ROC curve analysis supports the predictive value of the CBC-derived inflammation indexes for patients with metabolic syndrome, it cannot be considered as the only reliable test to confirm the clinical validity of these biomarkers. It would be beneficial to study other inflammation markers, such as cytokines and oxidation parameters, which were unfortunately not routinely determined in all our hospitalized patients.

Finally, it is noteworthy that our study was conducted within the Italian context, focusing on a population of severely obese subjects seeking an in-hospital multidisciplinary BWRP. For this reason, these results may be different from those obtained in populations with different degrees of obesity and residing in other countries.

In conclusion, this study underscores the validity of the CBC-derived inflammation indexes, easily obtainable routinely and at a low cost, in predicting MetS in individuals with severe obesity. Furthermore, the relationships between these indexes and the cardiometabolic risk factors can allow clinicians to establish the grading of the MetS associated with obesity in a better way.

## Data Availability

The datasets used and/or analyzed during the current study will be available on the Zenodo repository (http://www.zenodo.org) upon a reasonable request to the corresponding author.

## Abbreviations

MetS: metabolic syndrome; CBC: complete blood count; BMI: body max index; NHR: neutrophil to high-density lipoprotein; MHR: monocyte to high-density lipoprotein; LHR: lymphocyte to high-density lipoprotein; PHR: platelet to high-density lipoprotein; SII: Systemic Immune-Inflammation Index, SIRI: Systemic Inflammation Response Index; AISI: Aggregate Systemic Inflammation Index; HDL-C: high-density lipoprotein; HOMA-IR: Homeostatic Model Assessment for Insulin Resistance; TG/HDL-C: triglyceride/high-density lipoprotein cholesterol; FPG: fasting plasma glucose; BP: blood pressure; SBP: systolic blood pressure; DBP: diastolic blood pressure WC: waist circumference; TG: triglyceride; ROC, receiver operating characteristic; AUC, area under the curve; *p*, *p*-value.
